# The Amelioration of Detrimental Biochemical Anomalies by Supplementing B, C, and E Vitamins in Subjects with Type 2 Diabetes Mellitus May Reduce the Rate of Development of Diabetic Retinopathy

**DOI:** 10.1155/2022/3886710

**Published:** 2022-09-01

**Authors:** Subhasish Pramanik, Kaustav Banerjee, Lakshmi Kanta Mondal

**Affiliations:** ^1^Department of Endocrinology & Metabolism, Institute of Post Graduate Medical Education & Research and SSKM Hospital, Kolkata, 700020 West Bengal, India; ^2^Decision Sciences Area, Indian Institute of Management Lucknow, Uttar Pradesh 226013, India; ^3^Department of Ophthalmology, Regional Institute of Ophthalmology, Medical College Campus, Kolkata, 700073 West Bengal, India

## Abstract

Excessive intracellular glucose in insulin-independent tissues including nerve, nephron, lens, and retina invites mishandling of metabolism of glucose resulting in a background of increased oxidative stress, advanced glycation end products (AGE) formation, lipid peroxidation, and failure of antioxidant defense systems in type 2 diabetes mellitus (T2DM). All these detrimental biochemical anomalies ultimately attack biological membranes and especially capillary beds of the retina, resulting in the breakdown of the inner blood-retinal barrier and the initiation of diabetic retinopathy (DR). If these disarrays are corrected to a large extent, the development of DR can be avoided or delayed. In this prospective clinical trial, 185 patients with T2DM who received B vitamins, vitamin C, and vitamin E along with antidiabetic medication for five years demonstrated a slower rate of the development of DR and reduced abnormal biochemical mediators like reactive oxygen species (ROS), malondialdehyde (MDA), AGE, and vascular endothelial growth factor (VEGF) compared to 175 T2DM individuals who were treated with only antihyperglycemic drugs.

## 1. Introduction

Though duration and uncontrolled hyperglycemia are considered to be the contributors to the pathogenesis of DR, some individuals with T2DM keep away from the development of complications from this disease for a prolonged period [1, 2]. We have illustrated in our previous studies that DR is a two-stage disease where the initial unseen part of apoptosis of pericytes and endothelial cells is guided by toxic mediators of hyperglycemia-induced biochemical derangements, and the manifested stage from microaneurysms to neovascularization is dictated by an increased secretion of VEGF [3, 4].

Initial enormous intracellular glucose activates enhanced glycolysis and tricyclic acid (TCA) pathway as far as possible. All of the useful energy liberated during oxidation of nearly all of the carbohydrates is made accessible within the mitochondria as reducing equivalents (-H or electrons). Hypermetabolic retinal cells generate energy by reducing molecular oxygen to water. During this process, some amounts of partially reduced reactive oxygen forms are produced as an unavoidable byproduct of mitochondrial respiration. Some of these forms are liberated as free radicals that can damage lipids, proteins, and nucleic acids [5]. They are defined as ROS. Consequently, cells create a defense system to prevent injury caused by these free radical products. An imbalance between free radical generating and radical scavenging systems results in oxidative stress, a condition that is associated with endothelial injury and dysfunction [6, 7]. Enhanced glycolysis and TCA cycle loaded with excessive fuel cannot continue for an indefinite period. There must be gradual exhaustion of oxidized cofactors which are integral parts of these two essential metabolic pathways. Therefore, the sequential paths will follow increased anaerobic glycolysis resulting in lactate-induced lowering of cellular pH and a decrease in essential enzymatic activities. The other side of this pathway invites glutamate cascade leading to lipid peroxidation and lipid-derived free radicals. Above all, significant portions of unutilized glucose are forced to enter into nonenzymatic glycation end product formation which takes an important role in the pathogenesis of DR [8].

Among the different detrimental pathways regarding enhanced glycation, oxidative stress, and lipid peroxidation derived from chronic hyperglycemia, cells attempt to create a barrier or defense system to maintain normal physiology. Superoxide dismutase (SOD), glutathione reductase (GR), glutathione peroxidase (GPX), and reduced glutathione (GSH), which are the normal cellular protectors against the development of damaging toxicity of free radicals and lipid peroxides, are severely affected by advanced AGEs, MDA, and ROS [3, 4, 6].

Considering the above-mentioned detrimental biochemical pathways occurring in T2DM, we attempted to reduce the toxicity of these routes to prevent the development of DR by treating with antihyperglycemic drugs supplemented with B vitamins, which act as the source of nicotinamide adenine dinucleotide (NAD+); flavin adenine dinucleotide (FAD+), an inhibitor of glycation; and vitamin E (*α*-tocopherol) and vitamin C (ascorbic acid), which function as the inhibitor of lipid peroxidation and scavengers of free radicals. This prospective cohort study tests the effectiveness of alternative management of T2DM in preventing or delaying DR development.

## 2. Methodology

The study was conducted on consecutively selected individuals who were diagnosed with T2DM between March 2012 and December 2012 at the “Diabetic Clinic” of the Medical College, Kolkata, and referred to the “Retina Clinic” of the “Regional Institute of Ophthalmology” (RIO), Medical College, Kolkata, for an eye check-up. A trained ophthalmologist examined each subject, and those who did not have any clinical evidence of DR (DWR) were included in to the study. As the study attempted to determine if treating DWR patients with antioxidants (vitamins C and E), B vitamins, and antihyperglycemic drugs could reduce development rates of DR compared to treating them only with antihyperglycemic drugs, study subjects were divided into two groups, A and B, and followed up each one-year interval throughout the following five years. The number of subjects in each study group was estimated by using the following formula from Chow et al. [9].

(1) $$\begin{array}{c} n=\frac{{\left[Z_{\alpha /2}\sqrt{p_{0}\left(1-p_{0}\right)}+Z_{\beta }\sqrt{p_{1}\left(1-p_{1}\right)}\right]}^{2}}{{\left(p_{1}-p_{0}\right)}^{2}}. \end{array}$$

The sample size is based on the testing problem *H*_0_ : *p* = *p*_0_ against *H*_*a*_ : *p* = *p*_1_, where *p*_1_ ≠ *p*_0_. In our context, *p*_0_ is the cumulative five-year incidence of DR following standard treatment regime, and *p*_1_ is the expected cumulative five-year incidence of DR under the proposed treatment regime. Following Cikamatana et al. [10], we assume *p*_0_ = 22.2% while we expect *p*_1_ to be 12.2%, indicating that the proposed treatment regime may reduce the progression of DR by (*p*_0_ − *p*_1_) = 10%, the effect size for our study. Considering 5% level of significance (*α*) and 90% desired power (1 − *β*), the minimum size for each group was set as *n* = 183 at the baseline, considering 20% rate of attrition. Finally, 185 subjects in Group A and 175 subjects in Group B were enrolled at the baseline into the study.

Subjects with T2DM were diagnosed based on the guidelines of the American Diabetic Association [11]. The study was approved by the “Institutional Ethics Committee” of the RIO, Medical College, Kolkata (Ref. No. RIO/MC/KOL/NON-SPON/119/11-2011), and informed consent was collected from all the patients according to the declaration of Helsinki. Further, the trial was registered with the clinical trial registry of India at http://ctri.nic.in (CTRI/2011/091/000192). The inclusion criteria were (1) subjects with T2DM who can only be treated with oral antihyperglycemic medicines and expected to remain physically stable during the course of the study with such medication; (2) best-corrected visual acuity (VA) ≥ 6/9 in each eye with no DR; and (3) literate patients who could give informed written consent and could read the Snellen VA chart. The exclusion criteria were (1) presence of any types of DR with or without diabetic macular edema (DME) as determined by dilated fundoscopic examination, spectral-domain optical coherence tomography (SD-OCT), and digital fundus photography; (2) hyperglycemic individuals who needed insulin to manage their condition; (3) the presence of concomitant conditions such as branch retinal vein occlusion, Eales' disease, or glaucoma in the eyes could alter the normal occurrence of microvascular complications; and (4) patients suffering from hypertension, coronary artery diseases, or strong family history of coronary artery diseases, peripheral vascular disease, chronic infection, thromboembolic events, and urinary microalbumin > 300 mg/dl were some of the conditions considered as exclusion criteria for this study. The presence of any type of DR among study subjects was diagnosed according to the modified guideline of “Early Treatment of Diabetic Retinopathy Study (ETDRS)” [12].

### 2.1. Treatment Protocol

Among study groups, Group A was given standard doses of the following: (i) vitamin B containing thiamine mononitrate 10 mg, (ii) riboflavin 10 mg, (iii) nicotinamide 45 mg, (iv) pyridoxamine HCl 3 mg, (v) calcium pantothenate 50 mg, (vi) cyanocobalamin 15 mcg (Neurobion Forte, MERCK PHARMA), (vii) ascorbic acid 500 mg (Celin, GlaxoSmithKline Pharmaceuticals), and (viii) lastly, *α*-tocopherol 400 (Evion, MERCK LTD India) along with required oral antihyperglycemic drugs. Group B was treated with only oral antihyperglycemic drugs. General physical health and ocular condition were assessed at baseline and at 12-month interval using standard clinical procedures for evaluating vision and ocular pathology, including, (i) best-corrected visual acuity by Snellen's VA Chart; (ii) intraocular pressure by applanation tonometry; (iii) contrast sensitivity by “Rabin Contrast Sensitivity Chart”; (iv) dilated fundus examination by direct, indirect ophthalmoscopy and slit-lamp biomicroscopy with +90D lens; and (v) central retinal thickness measurement by spectral-domain optical coherence tomography (Spectralis, Heidelberg Engineering, Germany).

### 2.2. Biochemical Investigations

About 10 ml of venous blood sample was collected from each study subject after 12 hours of the overnight fast and was aliquoted in the following manner: 6 ml in the ethylenediamine tetraacetic acid (EDTA) and another 4 ml in the clot activating vials. From this 6 ml blood, 4 ml was taken in to a 15 ml sterile centrifuge tube for the isolation of peripheral blood mononuclear cells (PBMC), and another 2 ml sample containing EDTA vial was then centrifuged at 3000 revolutions per minute for 10 minutes at 4°C to separate plasma and cellular components. Buffy coat was removed by careful aspiration, and packed erythrocytes were then washed with cold phosphate-buffered saline (pH—7.2) by maintaining 4°C temperature for estimation of HbA1C (%), SOD, GR, and GSH activities. Plasma samples were collected in cryovials for the assessment of glucose. The serum sample was collected from the remaining 4 ml sample and preserved at -80°C for further estimation of AGE, MDA, and VEGF. The level of the ROS generation was investigated in the PBMC.

### 2.3. Measurement of ROS Generation in the PBMC

Intracellular ROS generation in PBMC cells (5 × 10^5^ pelleted cells) was measured by flow cytometric assay method [13] using a ROS-sensitive cell-permeable dye 2′ 7′ dihydrodichlorofluorescein diacetate (2′ 7′ H2DCFDA). In this method, 2′ 7′ H_2_DCFDA oxidized to highly fluorescent 2′ 7′-dichlorofluorescein (2′ 7′ DCF) presence of ROS in the PBMC. The PBMC exhibited an increased fluorescence of oxidized DCF, as measured by a flow cytometer (FACSCalibur, Becton Dickinson, San Jose, CA) fitted with an argon-ion laser (15 mW) set to a wavelength of 488 nm. The fluorescence of DCF was collected in FL1 channel, equipped with a 530/30 nm band-pass filter. Fluorescence was measured in the long mode using “Cell Quest Pro” software (BD Bioscience, San Jose, CA) and expressed as geometrical mean fluorescence channel (GMFC). Cells were gated on the basis of their characteristic morphology, i.e., forward scatter and side scatter of monocytes and lymphocytes. Acquisitions were performed on 10000 gated events; while data analysis was carried out with “Cell Quest Pro” software (BD Bioscience).

### 2.4. Measurement of MDA

The MDA content of the serum was measured by the thiobarbituric acid (TBA) assay method as described by Satoh [14]. In the assay, MDA reacts with TBA to produce a chromogenic adduct. The color product was measured using a spectrophotometer (Halo DB-20, Dynamica, Salzburg-Mayrwise, Austria) at 532 nm wavelength.

### 2.5. Measurement of SOD Activity

Erythrocyte SOD activity was measured using the kit of BioVision (catalog no. K335-100; Mountain View, CA 94043, USA). The SOD assay kit utilizes WST-1 solution and enzyme solution. WST-1 solution produces a water-soluble formazan dye upon reduction with superoxide anion. The rate of reduction with a superoxide anion is linearly related to the xanthine oxidase activity, which was used as an enzyme solution in this assay and was inhibited by SOD present in the sample. The inhibition activity of SOD was determined spectrophotometrically at a 450 nm filter using a microplate reader MerilyzerEiaquant (Meril Diagnostics Pvt. Ltd., Vapi, Gujarat). The activity of SOD was calculated according to the formula given below:

(2) $$\begin{array}{c} \text{SOD activity }\left(\text{inhibition rate}\%\right)=\frac{\left(A_{\text{blank}1}-A_{\text{blank}3}\right)-\left(A_{\text{sample}}-A_{\text{blank}2}\right)}{\left(A_{\text{blank}1}-A_{\text{blank}3}\right)}\times 100. \end{array}$$

### 2.6. Measurement of GR Activity

Erythrocyte GR activity was measured using the kit of BioVision (catalog no. K 761-200, 155 S. Milpitas Boulevard, Milpitas, CA 95035 USA). In the assay, GR reduces oxidized glutathione (GSSG) to reduced glutathione (GSH), which reacts with 5,5′-dithiobis (2-nitrobenzoic acid) (DTNB) to generate TNB2^−^. GR present in the sample generates yellow-colored TNB2 from DTNB. Absorbance of the color product was measured at the initial stage of reaction (first reading) and after 10 min (second reading) at 25°C of incubation at a 405 nm filter by using a microplate reader MerilyzerEiaquant (Meril Diagnostics Pvt. Ltd., Vapi, Gujarat). The GR activity detection range of the assay is 0.1–40 mU/ml. The amount of TNB2 (Δ*B*) produced by GR was determined by the TNB standard curve ranging from 0 to 50 nmol/well, and finally, the activity of GR was calculated using the following equation:

(3) $$\begin{array}{c} \text{GR activity}=\frac{∆B\times \text{sample dilution factor}}{\left(T_{2}-T_{1}\right)\times 0.9\times V}, \end{array}$$

where ∆*B* is the TNB amount from the TNB standard curve (in nmol), (*T*_2_ − *T*_1_) is the time difference of the first and second reading (in minute), *V* is the volume of pretreated sample added into the reaction well (in ml), and 0.9 is the sample volume change factor during sample pretreatment procedure.

### 2.7. Measurement of GSH

A hundred microliters of pelleted erythrocytes and an equal volume of 5% sulfosalicylic acid (SSA) solution were taken in a microcentrifuge tube and vortexed vigorously and kept on the ice for 10 minutes. The content of the tube was then centrifuged at 12000 × g and 4°C for 10 minutes. After, the supernatant was collected, it was diluted 10-fold and used for the measurement of GSH by using commercial kits of Sigma-Aldrich (catalog no. MAK364, St Louis, MO, USA). Assay kit was based on an enzymatic cycling method in the presence of GSH and a chromophore. The reduction of the chromophore produced a stable product, which was measured kinetically using a 450 nm (A450) filter in a microplate reader MerilyzerEiaquant (Meril Diagnostics Pvt. Ltd., Vapi, Gujarat). The absorbance is directly proportional to the amount of GSH in the sample. The assay was reproducible and able to detect a GSH concentration of 50 pmol/well in a 100-microliter reaction.

### 2.8. Measurement of the Serum AGE

The serum level of total AGE was measured by the competitive enzyme-linked immunosorbent assay (ELISA) method using the Cell Biolabs kits (Cat no. STA-817 Cell Biolabs, San Diego, CA, USA). The kit included an ELISA plate coated with AGE conjugate. The unknown samples or AGE-bovine serum albumin (BSA) standards were then added to the AGE conjugate preabsorbed ELISA plate. After a brief incubation, an anti-AGE polyclonal antibody was added, followed by an HRP-conjugated secondary antibody. Then, a color developed, and the absorbance of that color product was read at 450 nm as the primary wavelength using a microplate reader MerilyzerEiaquant (Meril Diagnostics Pvt. Ltd., Vapi, Gujarat) against the reduced BSA standard as the absorbance blank.

### 2.9. Measurement of Human Serum VEGF

The VEGF content in serum of each study subject was estimated by the ELISA method and using a commercially available kit (REY Biotech, Cat. No. ELH-VEGF-001, Norcross USA). The kit included an antibody specific to a human VEGF-coated well plate. The standards and samples were added into the wells. VEGF protein present in the sample was bound to the wells by the immobilized antibody. The wells were then washed several times, and a biotinylated anti-human VEGF antibody was added. Following a buffer wash, HRP-conjugated streptavidin was pipetted into the wells. Further, the wells were subjected to washing again. The TMB substrate solution was then added to wells and allowed to incubate for 30 minutes at room temperature. Then, a final color developed, whose intensity was proportional to the concentration of VEGF protein in the sample. The absorbance of the color product was measured colorimetrically by using a 450 nm filter in an ELISA plate reader MerilyzerEiaquant (Meril Diagnostics Pvt. Ltd., Vapi, Gujarat). The concentration of the VEGF in the sample was calculated using the standard curve and expressed in picograms per milliliter. The minimum detectable dose of VEGF in this method was <10 pg/ml. The intra- and interassay coefficient of variations (CV) for this method was <10% and <12%, respectively.

## 3. Statistical Analysis

We assume that 185 and 175 subjects in Group A and Group B are random samples from their respective populations. Baseline demographic, clinical, and biochemical parameters of two study groups were represented as mean ± SD (standard deviation) and compared by unpaired two-tailed Student's *t*-test. The baseline level of biochemical parameters in each group was compared with their levels after 5 years of treatment by paired two-tailed Student's *t*-test. Categorical data of two groups were presented as percentages and further evaluated by using the *Z* test. Pearson correlation coefficients were used to evaluate the association between two continuous variables. Survival analysis was performed to compare the progression rate of DR between two groups. Further, a Cox regression analysis was also conducted to assess factors that influence DR progression. A *p* value < 0.05 was considered statistically significant.

## 4. Results

**Figure 1:**
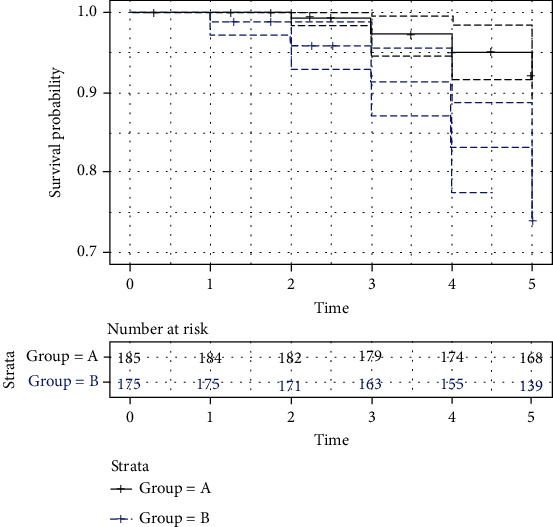
Kaplan–Meier survival analysis of progression to DR in Group A and Group B. The survival probability plot showed a significant difference (*p* value 2.21 × 10^−5^) in the two groups.

- Study results showed that 177 individuals (95.67%) in Group A and 168 individuals (96%) in Group B completed the 5-year follow-up period. Eight subjects (4.32%) from Group A and 7 (4%) subjects from Group B failed to attend the usual follow-up at each 1-year interval
- The comparison of the baseline demographic and clinical parameters like age, sex distribution, BMI, FPG, PPG, and HbA1c between the groups showed no significant differences (Table 1)
- Different biochemical parameters like PBMC ROS, MDA, GSH, SOD, GR, AGE, and VEGF demonstrated no significant differences when comparing A0 (baseline levels of different biochemical parameters of Group A) with A5 (levels of different biochemical parameters of Group A after five years) and B0 (baseline levels of different biochemical parameters of Group B), respectively. However, B5 (levels of different biochemical parameters of Group B after five years) showed significantly higher levels of PBMC ROS, MDA, AGE, and VEGF and lower levels of GSH, SOD, and GR compared to B0 (Table 2)
- The correlation analysis revealed a significant positive correlation of VEGF with PBMC ROS, MDA, and AGE and a negative correlation with GSH, SOD, and GR levels for A0, B0, and B5, respectively. Hence, for A5, the study demonstrated only a significant negative correlation between VEGF and GR (Table 3)
- The Cox regression model demonstrated that treating patients with only oral antidiabetic medication increases the hazard of DR by a factor of 3.795, (95% LCL = 2.08, UCL = 6.93): keeping other predictors constant, without vitamin B, C, and E supplements, the risk of DR is increased by 279%. Similarly, for one-unit increase in VEGF, the risk of DR is increased by 3.6% (95% LCL = 1.01, UCL = 1.07) and a unit increase in MDA increases the risk of DR by a factor of 7.5% (95% LCL = 0.71, UCL = 1.64) (Table 4). Further, survival probability plot exhibited a significant difference (*p* value of the log-rank test: 2.21 × 10^−5^) between the two groups (Figure 1)
- There were no adverse effects of B vitamins, vitamin C, or vitamin E supplementation reported during the study

## 5. Discussion

It has been demonstrated by landmark studies that improving glycemic control reduces the risk of development and progression of DR, though there is no lower limit of glycemic control that is protective against the onset of retinopathy [15, 16]. Our sequential studies have suggested that hyperglycemic and dyslipidemia-induced biochemical derangements result in tissue hypoxia and upregulation of VEGF [17, 18]. The important anomalies related to mishandling of glucose and lipids leading to increased generation of advanced glycation and lipid peroxidation end products, oxidative stress, and failure of the body's antioxidant defense systems ultimately invite inflammation, hypoxia, and increased VEGF secretion [17, 18].

This study included 185 diabetic patients who had been treated with antihyperglycemic medication along with vitamins B, C, and E since their diagnosis in the year 2012 and 175 patients who were treated only with antihyperglycemic medications. Conventional control of hyperglycemia with average HbA1c between 7 and 7.5% did not illustrate any significant visual disturbance and microangiopathy on dilated fundus examination and 3-D spectral OCT. It is hypothesized that niacin and riboflavin administered exogenously provide the steady supply of NAD+, FAD+, and flavin mononucleotide (FMN), the most important oxidized cofactors for continuity of tricarboxylic acid cycle (TCA) and glycolysis. Energy liberated from oxidation of all food stuffs generates reducing equivalents that are directly collected and carried to mitochondrial respiratory systems to reduce oxygen, yielding adenosine triphosphate (ATP), water, and some unavoidable byproducts such as ROS, which are not only detrimental to essential cellular components but also responsible for diminished activities of oxidant scavenging enzymes like SOD and GR.

Administration of ascorbic acid, *α*-tocopherol, and B vitamins containing thiamine, niacin, riboflavin, pyridoxamine, and pantothenic acid eliminates inhibition of glycolysis and TCA cycle and reduces lipid peroxidation and free radical accumulation. Two experimental animal studies have demonstrated beneficial effects of vitamin thiamine and niacin to ameliorate different detrimental pathways related to hyperglycemia [19, 20]. Another groundbreaking animal model diabetic study by Kowluru et al. revealed that a diet supplemented with multinutrients like ascorbic acid, cholecalciferol, d-alpha tocopherol, Fish Oil EE 70%, eicosapentaenoic acid, docosahexaenoic acid, benfotiamine, *α* lipoic acid, tocomin, zeaxanthin, lutein, and proprietary blend containing Polygonum cuspidatum SE (resveratrol), green tea, turmeric root (curcuminoids), N-acetyl-cysteine, Pycnogenol® pine bark, grape seed extract, coenzyme Q10 and zinc, and soybean oil prevents DR and also maintains normal retinal function, mitochondrial homeostasis, and inflammatory mediators [21]. Inhibition of AGE formation and accumulation by pyridoxamine has been tested in an experimental diabetic model [22]. In our previous study, we also demonstrated the effectiveness of vitamin supplements like B, C, and E in reducing oxidative stress-induced structural and functional abnormalities of red blood cells (RBCs) in preventing DR development [23].

The present study clearly demonstrates that regular intake of niacin, riboflavin, thiamine, pyridoxal phosphate, ascorbic acid, and *α*-tocopherol reduces the circulatory levels of surrogate markers of lipid peroxidation (MDA), ROS, and VEGF and, on the contrary, increases serum level of intracellular antioxidants, SOD, GR, and GSH. GR is an ever-present enzyme that reduces oxidized glutathione (GSSH) to reduced forms of GSH which is an omnipotent intracellular antioxidant [24]. Another study has suggested that decreased activity of superoxide dismutase is associated with microvascular complications in T2DM individuals [6].

Nonenzymatic glycation end products and excessive free radicals may alter the conformation and activities of cellular antioxidant enzymes SOD and GR, whereas vitamins C and E and pyridoxamine might attenuate these injurious pathways and rid the cellular system of malfunctioning. Riboflavin and niacin, the precursors of FAD+ and NAD+, the important electron carriers in mitochondria, for oxidative phosphorylation, help the continuity of glycolysis and TCA at a steady state.

It is hypothesized from this prospective study that glucose metabolism machinery requires a sufficient supply of NAD+ and flavoproteins, FAD+, and FMN to run the glycolysis and TCA cycle. Prevention of nonenzymatic glycation which disturbs the normal function of proteins by cross-linking, disulfide bond formation, and chemical rearrangement is very essential to stop or delay diabetic microvascular complications. Stoppa et al. demonstrated that reduced antioxidant enzyme activity owing to hyperglycemia is lessened by aminoguanidine [25].

A similar detrimental pathway of glutamate-induced increased intracellular Ca^++^-mediated lipid peroxidation is believed to take an important part in the pathogenesis of the development of diabetic complications [8, 26]. Regular intake of vitamin E inhibits lipid peroxidation and production of MDA which are toxic to capillary endothelium [27]. Consequently, oxidative stress, i.e., increased formation of free radicals due to increased TCA cycle and lipid peroxidation, is considered a crucial player in the diminished ability of the intracellular antioxidant defense system and invitation of proinflammatory and inflammatory cytokines [28]. As the involvement of retinal vascular endothelial cell dysfunction in the pathogenesis of DR is suggested by AGE activation, ROS generation, and lipid peroxidation, systemic administration of antioxidants like vitamin C, vitamin E, and pyridoxal phosphate (vitamin B6) may be adequate to suppress these detrimental pathways. Isolated experimental studies have highlighted the efficacy of vitamins B1, B3, and B6 to prevent diabetes-induced retinal vascular lesions, whereas complex multiple pathways derived from hyperglycemia-induced biochemical derangements need multiple blockers.

The novelty of this study is the addition of attention beyond AGE formation and ROS production in diabetes mellitus, i.e., assistance in the uninterrupted running of glycolysis and Krebs cycle and pentose phosphate pathway to generate adequate ATP and intracellular antioxidant defense.

As the “Age-Related Eye Disease Study” suggested that a nutritional supplement could prevent the progression of age-related macular degeneration [29], we took this clinical trial to evaluate initially the efficacy of vitamins B, C, and E to ameliorate the detrimental pathways involved in the development of DR in T2DM. A large multicenter, double-blind randomized controlled clinical trial is required to validate these findings.

## 6. Conclusion

The present study demonstrated that vitamin B, C, and E supplements in combination with conventional management of hyperglycemia decrease the risk of development of DR by inhibiting oxidative stress, AGE formation, lipid peroxidation, and VEGF secretion. This finding may suggest a new approach to DR management.

## Figures and Tables

**Table 1:** Baseline demographic and clinical characteristics of study subjects of two groups.

Parameters	Group A0	Group B0	*p* value
Age (years)	54.6 ± 8.96	54.6 ± 9.12	0.962
Sex (M/F)	117/68	105/70	0.527
BMI (kg/m^2^)	24.63 ± 4.46	24.57 ± 4.49	0.904
FPG (mg/dl)	126.55 ± 39.08	128.58 ± 42.34	0.637
PPG (mg/dl)	264.62 ± 74.72	267.06 ± 78.82	0.764
HbA1c (%)	7.85 ± 1.75	7.95 ± 1.71	0.647

Group A0: baseline levels of different parameters of Group A; Group B0: baseline levels of different parameters of Group B; FPG: fasting plasma glucose; PPG: postprandial plasma glucose; HbA1c: glycated haemoglobin. Continuous observations were presented as mean ± SD (standard deviation) and compared by using the unpaired *t*-test. Categorical data of the two groups were presented as ratios and compared using the *Z* test; *p* value < 0.05 was considered statistically significant.

**Table 2:** Comparison of different biochemical parameters between the groups at baseline and within the groups after 5 years.

Parameters	Group A0	Group B0	*p* value for A0 vs. B0	A5	*p* value for A0 vs. A5	B5	*p* value for B0 vs. B5
PBMC ROS (geomean of DCF/10^5^ cells)	97.35 ± 11.31	97.61 ± 14.38	0.846	96.3 ± 15.98	0.539	101.0 ± 14.95	0.026
MDA (nmol/ml)	2.59 ± 0.57	2.63 ± 0.61	0.569	2.56 ± 0.89	0.804	2.89 ± 0.84	0.0006
SOD (U/ml)	42.10 ± 3.39	42.06 ± 3.13	0.891	42.61 ± 3.96	0.337	40.84 ± 3.56	0.0003
GR (mU/ml)	23.12 ± 3.64	23.09 ± 3.31	0.946	23.26 ± 3.97	0.701	22.0 ± 3.25	0.0009
GSH (nmol/ml)	26.14 ± 5.18	26.34 ± 4.94	0.699	26.27 ± 4.76	0.546	21.41 ± 4.15	<2.2 × 10^−16^
AGE (*μ*g/ml)	3.09 ± 0.62	3.12 ± 0.71	0.699	3.09 ± 0.79	0.938	3.53 ± 1.04	3.15 × 10^−5^
VEGF (pg/ml)	96.5 ± 9.78	96.7 ± 9.31	0.841	96.4 ± 11.8	0.921	99.5 ± 9.15	0.0002

A0: baseline levels of different parameters of Group A; A5: levels of different parameters of Group A after 5 years; B0: baseline levels of different parameters of Group B; B5: levels of different parameters of Group B after 5 years; PBMC ROS: peripheral blood mononuclear cell reactive oxygen species; MDA: malondialdehyde; SOD: superoxide dismutase; GR: glutathione reductase; GSH: reduced glutathione; AGE: advanced glycation end products; VEGF: vascular endothelial growth factor. Two sets of paired observations were compared by paired *t*-test; *p* value < 0.05 was considered statistically significant.

**Table 3:** Correlation of VEGF with different biochemical parameters (represented with correlation coefficient “*r*” with “*p* value”) at baseline and after 5 years of two study groups.

Group	MDA	PBMC ROS	SOD	GR	AGE	GSH
A0	0.40 (1.61 × 10^−8^)	0.321 (8.13 × 10^−6^)	-0.262 (0.0003)	-0.223 (0.002)	0.262 (0.0003)	-0.222 (0.002)
A5	0.14 (0.059)	0.098 (0.195)	-0.065 (0.388)	-0.155 (0.039)	0.12 (0.112)	-0.13 (0.087)
B0	0.239 (0.001)	0.318 (1.81 × 10^−5^)	-0.275 (0.0002)	-0.187 (0.013)	0.257 (0.0006)	-0.213 (0.005)
B5	0.405 (5.23 × 10^−8^)	0.398 (8.96 × 10^−8^)	-0.388 (2.05 × 10^−7^)	-0.354 (2.54 × 10^−6^)	0.342 (5.67 × 10^−6^)	-0.306 (5.44 × 10^−5^)

Pearson's correlation was used, and *p* value < 0.05 was considered statistically significant.

**Table 4:** Cox regression showing variables that influence the development of DWR to DR.

Variables	Coef	Exp (coef)	SE (coef)	*Z*	*p* value
Group B	1.334	3.795	0.308	4.336	1.45 × 10^−5^
VEGF	0.036	1.036	0.015	2.329	0.0199
MDA	0.072	1.075	0.214	0.337	0.7359

MDA: malondialdehyde; VEGF: vascular endothelial growth factor. The best three-predictor model was selected by carrying out forward and backward stepwise regression procedure. The final model demonstrated that treating patients with only oral antidiabetic medication (Group B) increases the hazard of diabetic retinopathy (DR) by a factor of 3.795, (95% LCL = 2.08, UCL = 6.93): keeping other predictors constant, without vitamin B, C, and E supplements, the risk of DR is increased by 279%. Similarly, for one-unit increase in VEGF, the risk of DR is increased by 3.6% (95% LCL = 1.01, UCL = 1.07) and a unit increase in MDA increases the risk of DR by a factor of 7.5% (95% LCL = 0.71, UCL = 1.64). All the asymptotically equivalent tests (likelihood ratio, Wald, and score tests) unanimously rejected the omnibus null hypothesis that all the regression coefficients are zero with *p* value ≤ 2 × 10^−5^.

## Data Availability

The data presented in this study are available on a reasonable request from the corresponding author.
